# Penetration depth study of 830 nm low-intensity laser therapy on living dog tissue

**DOI:** 10.14202/vetworld.2020.1417-1422

**Published:** 2020-07-23

**Authors:** Naruepon Kampa, Supranee Jitpean, Suvalak Seesupa, Somphong Hoisang

**Affiliations:** 1 Division of Surgery, Faculty of Veterinary Medicine, Khon Kaen University, Khon Kaen, 40002, Thailand; 2 Veterinary Teaching Hospital, Faculty of Veterinary Medicine, Khon Kaen University, Khon Kaen, 40002, Thailand

**Keywords:** living dog tissue, low-intensity laser therapy, mean output power, penetration depth

## Abstract

**Background and Aim:**

Recent studies have shown that low-intensity laser therapy (LILT) enhances chronic wound healing, reduces pain, reduces inflammation, and improves post-operative rehabilitation. However, clinical outcomes in the veterinary use of LILT vary between different experimental studies. This is explained by improper laser parameter settings and limits of its penetration depth. This study aimed to investigate the penetration depth of 830 nm LILT on living dog tissue in different operating modes. This entailed continuous wave (CW) versus pulse wave (PW) and with contact versus non-contact techniques of the laser probe at different tissue-laser probe distances. The results can be applied for use in clinical practice.

**Materials and Methods:**

Twenty-four dogs that had undergone abdominal surgery were included in this study. The laser parameters were set at 200 mW, fluence of 4 J/cm^2^ and the laser power output denoted as mean output power (MOP) was measured by a power meter.

**Results:**

The MOP of the 830 nm CW laser was significantly higher than the PW laser (p<0.05). The MOP of the contact technique was significantly greater than that of the non-contact technique in both CW and PW modes (p<0.05). The MOP through the skin tissue was between 16.09 and 18.60 mW (8.05-9.30%) for the contact technique and 8.73 and 19.36 mW (4.37-9.68%) for the non-contact technique. In the muscle-skin layer, the MOP was between 0.50 and 1.56 mW (0.25-0.78%) and the MOP was not detected using the non-contact technique with a 5 cm tissue-laser probe distance.

**Conclusion:**

Our study indicates that 830 nm LILT (with laser parameter setting at 200 mW, fluence of 4 J/cm^2^ for both contact and non-contact techniques, and tissue-laser probe distance up to 5 cm) was appropriate for treatments within 14 mm of depth. However, the use of 830 nm LILT for an application in which the target tissue is deeper than 14 mm may limit its positive effect.

## Introduction

Low-intensity laser therapy (LILT), known as photobiomodulation therapy, stimulates the mitochondrial respiratory chain and causes changes in cellular adenosine triphosphate or cyclic adenosine monophosphate levels [1]. Recent studies suggest that LILT can be used to enhance chronic wound healing in rats [2,3], to reduce pain in dogs [4] and humans [5], to reduce inflammation in rats [3,6], and to improve post-operative rehabilitation in dogs [7] and humans [8]. Nonsteroidal anti-inflammatory drugs (NSAIDs) are widely used worldwide to reduce wound pain. However, a recent study indicated that NSAIDs could affect the wound healing process by reducing tissue integrity [9]. For this reason, using LILT as alternative treatment for wound healing will be beneficial because LILT both reduces pain and enhances the wound healing. In addition, the positive effects of LILT in companion animals have been demonstrated for canine osteoarthritis [4], for stimulating hair regrowth in dogs with non-inflammatory alopecia [10], and for the treatment of pyogranulomatous pododermatitis in dogs [11]. However, several studies in veterinary medicine have not shown any beneficial outcomes. Studies have used LILT for a variety of purposes. For instance, Kurach *et al*. [12] applied 635 nm LILT with fluence of 1.125 J/cm^2^, 3 times weekly on open wounds in healthy dogs and there were no beneficial effects on wound healing after the treatment for 1 month. A study by Bennaim *et al*. [13] reported that using 810 nm LILT, fluence of 329.7 J/cm^2^, power density of 5.5 W/cm^2^, and frequency of 2.5 Hz over hemilaminectomy for 1 min in dogs had no significant difference in many parameters compared to a control group. This includes recovery time, duration of post-operative pain management, surgical outcomes, and adverse effects. In another study, Gammel *et al*. [14] reported that irradiation of 980 nm LILT with fluence of 5 J/cm^2^ once a day for 5 days did not enhance wound healing of small incisions and uncomplicated wounds in dogs. Recently, in a study by Kennedy *et al*. [15], the 635 nm LILT was irradiated with a fluence of 1.5-2.25 J/cm^2^ for 5 min, over 10 sessions within 96 h after tibial plateau leveling osteotomy in dogs. After this intervention, there were no beneficial effects on pain reduction and hindlimb function.

In small animal practice, the non-beneficial results may be either affected or caused by the different laser treatment parameters, including laser class, laser wavelength, laser operation mode (pulse wave [PW] or continuous wave [CW]), energy density, power density, exposure time, frequency, and number of treatment sessions [16]. Moreover, the differences in skin thickness and skin characteristics in different species may affect laser-tissue interaction [17]. In dogs, the skin thickness measured using ultrasonography has a mean thickness within 0.97-3.6 mm [18,19], whereas mean skin thickness in humans is within 2.5-5.0 mm [20,21]. Therefore, the beneficial outcomes in veterinary practice may require proper parameter settings and an understanding of the limitations of the LILT equipment.

The aim of the present study was to investigate the penetration depth of 830 nm LILT on living dog tissue in different operation modes (CW vs. PW, with contact vs. non-contact techniques of laser probe use) and at different tissue-laser probe distances.

## Materials and Methods

### Ethical approval

The study was approved by the Institutional Animals Care and Use Committee of Khon Kaen University (IACUC-KKU 37/60).

### Samples

Twenty-four mixed breed dogs averaging 16.34 kg body weight with normal appearance on physical examination and with normal blood profiles were included in the study. All dogs underwent abdominal surgery for conventional ovariohysterectomy.

### Experimental protocols and measurements

The dogs were administered with 0.2-0.3 mg/kg of diazepam intravenously (IV) and 0.3-0.5 mg/kg of morphine sulfate intramuscularly. The surgical site was clipped and the tissue thickness was measured in each dog using ultrasonography (Logic^®^ S7, GE, USA) with a frequency of 10 MHz and a broad-spectrum linear probe (9L-D) by the same operator. A cephalic IV catheter was placed, and lactated Ringer’s solution was given at 10 ml/kg/h. Each dog was anesthetized using thiopental sodium 10 mg/kg IV and maintained with isoflurane 1-2% in pure oxygen.

**Figure-1:**
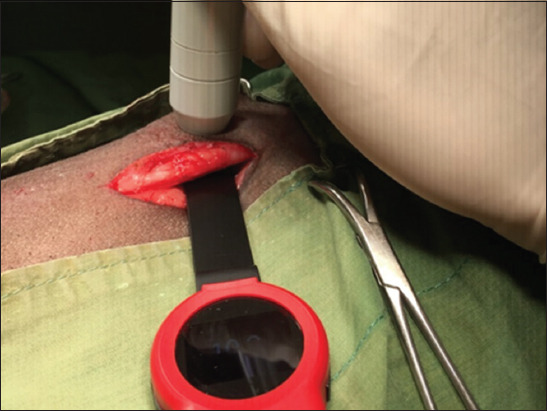
Demonstrating the power meter placement under skin-muscle tissue and measuring mean output power.

**Figure-2:**
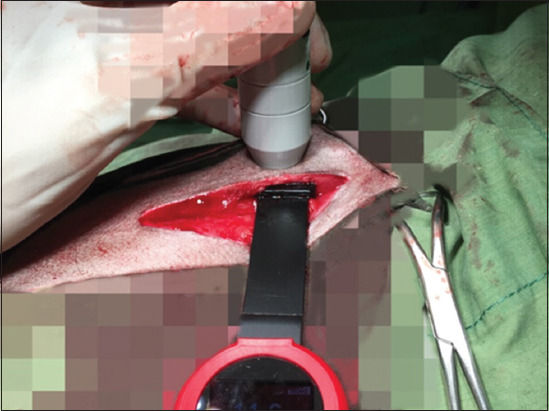
Measuring the mean output power using a power meter inserted inside the tissue.

**Figure-3:**
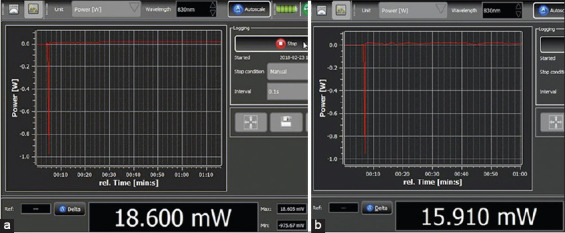
An example of mean output power (MOP) measured by Thorlabs power meter software through skin tissue by skin contact (a) with continuous-wave (CW) mode and (b) with pulse wave (PW) mode; MOP with CW mode in the same tissue was higher than with the PW mode.

The 830 nm diode LILT (Class 3B laser: BTL 5000 series, BTL Industries Ltd., UK) was set at a power of 200 mW as in the previous studies [22,23]. The radiant exposure was set at 4 J/cm^2^ which has been recommended for the treatment of inflammation, wounds, and superficial pain [24]. Both CW and PW modes with frequency of 50 Hz were irradiated for 60 s over the power meter without tissue (air) as the control and with tissues (skin-muscle tissue and skin) (Figures-1 and 2). The power meter (PM160T, Thorlabs^®^, USA) was used to measure the laser power density, which was represented by MOP. The laser probe was held above the tissue surface at different distances at 0 (contact), 1, and 5 cm tissue-laser probe distances. The MOPs were recorded at 10, 20, 30, 40, 50, and 60 s using Thorlabs power meter software (Figure-3a and b).

### Statistical analysis

Descriptive analysis represented power penetration as mean (mean output power [MOP]) and standard deviation. The effect of laser operation mode (CW vs. PW), tissue thickness (air, skin, and skin-muscle), and tissue-laser probe distance on MOP was tested by three-way factorial analysis of variance (ANOVA) with blocking (dogs). If the overall effect was significant, we proceeded to do a one-way ANOVA analysis of the effect of laser operation and tissue-laser probe distance in each tissue thickness. All significant differences of the tests were considered significant if p<0.05.

## Results

Our data showed no significant difference between tissue sample (dogs) and no effect on MOP. The MOP of 830 nm LILT without tissue (control) was significantly different between laser operation modes (p<0.05). Without any distance between probe and power meter (0 cm, contact technique), the MOP was 144.53 mW (72.27%) and 118.93 mW (59.47%) in CW and PW modes, respectively. There was a significant difference of MOP at 1 cm power meter-laser probe range compared to the contact technique (p<0.05). However, there was no difference between 1 cm and 5 cm power meter-laser probe range in both CW and PW modes, as shown in Table-1.

**Table-1:** Comparison of MOP between the use of 830 nm CW and PW in live dog tissue in different situations, including without tissue (air) as the control and with tissues (skin-muscle tissue and skin).

Tissue (thickness)	Tissue-laser probe distance	MOP (mW)	

		830 nm LILT (CW) % (MOP±SD)	830 nm LILT (PW, 50 Hz) % (MOP±SD)
Air (control: 0 mm)	Contact	72.27 (144.53±7.87)	59.47 (118.94±6.33)
	1 cm	64.49 (128.98±17.81)	51.98 (103.95±9.42)
	5 cm	61.38 (122.76±9.52)	50.55 (101.09±8.22)
Skin (2.9 mm)	Contact	9.30 (18.60±7.11)	8.05 (16.09±8.28)
	1 cm	9.68 (19.36±9.87)	5.93 (11.85±5.87)
	5 cm	6.73 (13.45±7.02)	4.37 (8.73±5.06)
Skin-muscle (14.38 mm)	Contact	0.78 (1.56±2.02)	0.50 (1.0±1.08)
	1 cm	0.25 (0.50±0.87)	0.34 (0.68±0.79)
	5 cm	Not detect	Not detect

MOP=Mean output power, LILT=Low-intensity laser therapy, CW=Continuous wave, PW=Pulse wave, SD=Standard deviation

By determining MOP in living dog tissue, it was significantly higher in the CW mode compared to the PW mode (p<0.05). The MOP through only skin tissue (2.9 mm thickness) was found to be 18.60 mW (9.30%) and 16.09 mW (8.05%) in CW and PW modes by contact technique, respectively. For a 1 cm tissue-laser probe distance (non-contact technique), there was significantly less MOP in PW mode (p<0.05). However, there was no difference of MOP in CW mode compared to the contact technique. For a 5 cm tissue-laser probe distance, a significant difference was observed between 1 cm and 5 cm tissue-laser probe distances in both CW and PW modes (p<0.05).

With the contact technique, the MOP through skin-muscle tissue (14.38 mm thickness) was found to be 1.55 mW (0.78%) and 1.00 mW (0.50%) in CW and PW modes, respectively. At a 1 cm tissue-laser probe distance, MOP was 0.49 mW (0.25%) in CW mode and significantly different compared to the contact technique (p<0.05). However, we were unable to detect the laser power with a 5 cm tissue-laser probe range, as shown in Table-1.

## Discussion

Penetration depths of LILT have been reported on skin flaps [23,25], dog cadaver [26], living experimental animals [16], and humans [22]. Our report was the first study on penetration depth of 830 nm LILT with 200 mW and fluence of 4 J/cm^2^ on living dogs. Measuring laser power through tissue is based on the principles of light detection. Spectrometers, photodiodes, power meters, thermopiles, and charge-coupled device cameras can be used to measure laser power. Our study used a PM160T power meter, which can directly measure the laser power, and is widely used in LILT studies [27]. The equipment consists of a sensor with an aperture of 1 cm^2^ that can detect light with optical power ranging from 100 μW to 2 W and over a wavelength ranging from 190 to 10,600 nm. Therefore, this power meter is suitable for measuring the LILT penetration depth of living tissue at a wavelength of 830 nm in this study.

**Figure-4:**
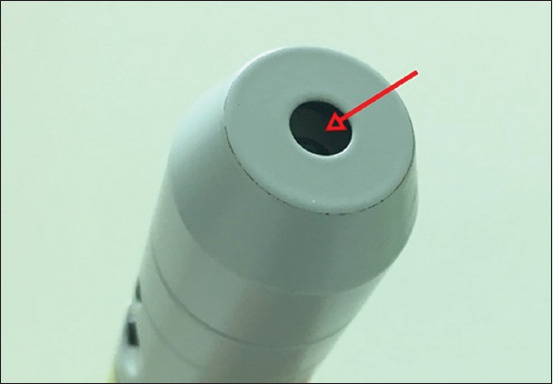
The converging probe for 830 nm with a hollow tip (arrow) and a small distance between incident light and the detector.

**Figure-5:**
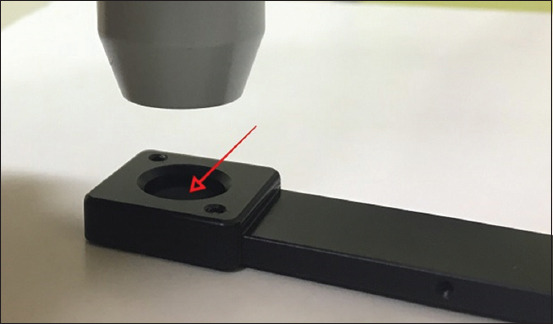
The PM160T power meter consists of an ultra-slim thermal sensor with concave power detector tip (arrow).

Our study demonstrated that some parts of the laser power from the initial setting were lost before the power meter. This loss could be due to the internal reflection of light according to the angle of incidence [28]. Moreover, the small distance between incident light, the detector of the hollow tip of the laser probe (Figure-4), and concave power detector tip (Figure-5) may lead to a loss of laser power as well. Therefore, it should be considered that the laser power at the target tissue may not be equal to that of the initial setting. In addition, different laser parameters have a strong effect on laser power in living tissue.

Regarding the operation mode, the MOP of the CW mode was obviously greater than the PW mode. This result is consistent with a study on the penetration of LILT in anesthetized rabbits [16]. Thus, the CW mode of LILT is recommended for the treatment of deeper organs excluding thermal side effects [29]. Regarding the operational technique in our study, the contact irradiation technique showed a significant difference compared to the non-contact technique (p<0.05). In another study, the contact technique with whole compression or partial compression of the laser probe showed improved light propagation [30]. For the non-contact technique without tissue, there was no difference between 1 cm and 5 cm tissue-laser probe range in both CW and PW modes. Therefore, LILT of deeper tissue should be performed by the contact technique, such as in post-operative rehabilitation purposes. LILT of superficial tissue can be performed by the non-contact technique with a 1-5 cm tissue-laser probe distance. However, when using LILT without direct contact, power can be lost due to reflection and scattering, making it possibly harmful to the operator.

In this study, the average skin thickness of the dogs was 2.9 mm, which correlates with a previous study [18]. The MOP through the skin was detected at 8.73-19.36 mW, about 4.37-9.68% of the initial setting (200 mW). In a previous study using 810 nm LILT CW, 200 mW power, and power density of 6.37 W/cm^2^ (parameters similar to our study), the MOP through euthanized rat skin flaps was higher than that in our study (39.0-40.8 mW [19.5-20.4%] with the contact technique and 46.2-51.0 mW [23.1-25.5%] with the non-contact technique) [23]. The difference in the results may be due to differences in skin thickness, with rat skin thickness at 1.1-1.3 mm, consequently affecting laser transmission [31]. According to laser-tissue interaction, blood, water, and melanin are the major absorbing components in living tissues [28]. Therefore, MOP detection in living tissue would be less when compared to cadaver tissue samples.

A clinical study in humans (using 810 nm CW and 200 mW) reported that the MOP of laser through the Achilles tendon with approximately 2.9 mm thickness was less than that in our study. The MOP was detected at 0.05 mW (0.025%) in the stretched state and 0.33 mW (0.17%) in the relaxed state [22]. The difference in results may be due to the double skin layer absorbing more light and containing more melanin which absorbs more laser photons [32]. In addition, Kolárová *et al*. [25] reported transmission of laser at 632.8 nm and 50 mW through a human abdominal skin flap of 2.6 mm thickness, where the MOP was found at 3.25 mW (6.5%). When laser light at 675 nm and 21 mW was used, the MOP was detected at 3.21 mW (15.3%). Our study used different laser wavelength and treatment parameters (830 nm and 200 mW) and that needs to be considered. Enwemeka [16] indicated that the transmission of longer laser wavelengths was greater than for shorter laser wavelengths.

The MOP through the muscle-skin tissue (14.38 mm thickness) can be detected between 0.5 and 1.56 mW (0.25-0.78%) using contact and non-contact techniques at a 1 cm tissue-laser probe distance. With 5 cm tissue-laser probe distance, no MOP was detected. Our data showed that the laser power of LILT was attenuated and absorbed in superficial tissue (and deeper tissue up to 14 mm) in living dogs. This result is in line with a study on dog cadavers by Piao *et al*. [26]. It was done with irradiation of 980 nm laser, 3.14 W/cm^2^ at T13 and reported a MOP at 0.04-0.23 mW/cm^2^. However, this study did not report the exact tissue thickness between the dog’s skin and the spinal cord. The animal’s anatomy, including skin barrier, heavy muscle cover, and thick skeletal bone may be an impediment to the potential penetration of LILT. Small laser power was found through the muscle-skin tissue. Theoretically, the beneficial positive effects of LILT are assumed to occur when the light photon is absorbed in tissue cells. This study showed that light photons were absorbed by target tissue from the skin surface. For therapeutic reasons, target tissue deeper than 14 mm should be used with caution and the laser parameters determined in advance before intervention with animals.

Several recent studies have reported that superpulsed wavelength laser (SPW) (the new technology of LILT) can deliver to deeper organs in living tissue [22,23]. Unfortunately, we faced the limitations of our power detector. The PM160T detected light optical power between 100 μW and 2 W within the wavelength range of 90 nm-10,600 nm and was not suitable for detecting superpulsed laser in living tissue. Based on our unpublished data, it was found that superpulsed multiple wavelengths (SPMW) (905, 860, and 660 nm) laser transmitted laser power greater than the 830 nm LILT in nine dog cadaver samples when using a S245C-L power detector (Thorlabs, USA). This detector can detect light optical power from 2 mW to 50 W within the wavelength range of 190 nm-20 μm. However, the S245C-L power detector is too large to use in living dog tissue.

Further studies are recommended to focus on the translation and clinical evaluation of 830 nm LILT parameters for the treatment of various tissue conditions in dogs such as chronic skin wounds, superficial pain, and deeper tissue injuries. In addition, SPMW should be evaluated for its penetration properties in small animal tissue for optimal treatment in veterinary practice.

## Conclusion

Our study revealed that 830 nm LILT setting at 200 mW, fluence of 4 J/cm^2^ in both CW and PW modes, can be used for superficial conditions with contact and non-contact techniques with tissue-laser probe distance up to 5 cm. Hence, laser therapeutic applications for deep tissue should apply the contact technique to improve light propagation. The tissue-laser probe distance at 5 cm should not be used in muscle-skin tissue as in this study. LILT in veterinary practice with target tissue deeper than 14 mm should be used with caution and the laser parameters checked in advance before intervention in animals.

## Authors’ Contributions

NK and SJ designed and supervised the study. SH conducted the literature search and performed the experiments. SS performed the data analysis. NK, SJ, and SH wrote the manuscript. All authors read, revised, and approved the final manuscript.
